# Supplementary material for: Effects of Environmental Chemical Pollutants on Microbiome Diversity: Insights from Shotgun Metagenomics
Source: Toxics. 2025 Feb 19;13(2):142. doi: 10.3390/toxics13020142 (PMC11861561; doi:10.3390/toxics13020142)
Supplement: Supplementary file 1 [file toxics-13-00142-s001.zip › toxics-3447975 Supplemental Materials Table S2 Figure S1.pdf]

Table S2: Names and concentrations of chemical pollutants at each collection site.

	Site	ReadCounts_SED_name	ReadCounts_WS_name
1	KKL	20476097.33	34616707.33
2	MEC	30385105.33	33736439.67
3	MEF	31737857	21904403.33
4	MET	41576421.67	32134992.33
5	MIE	18537225.33	28940417.67
6	MIM	22971062.33	25820810.67
7	MIP	22941852	28089634
8	UCJ	35277928.33	25489005

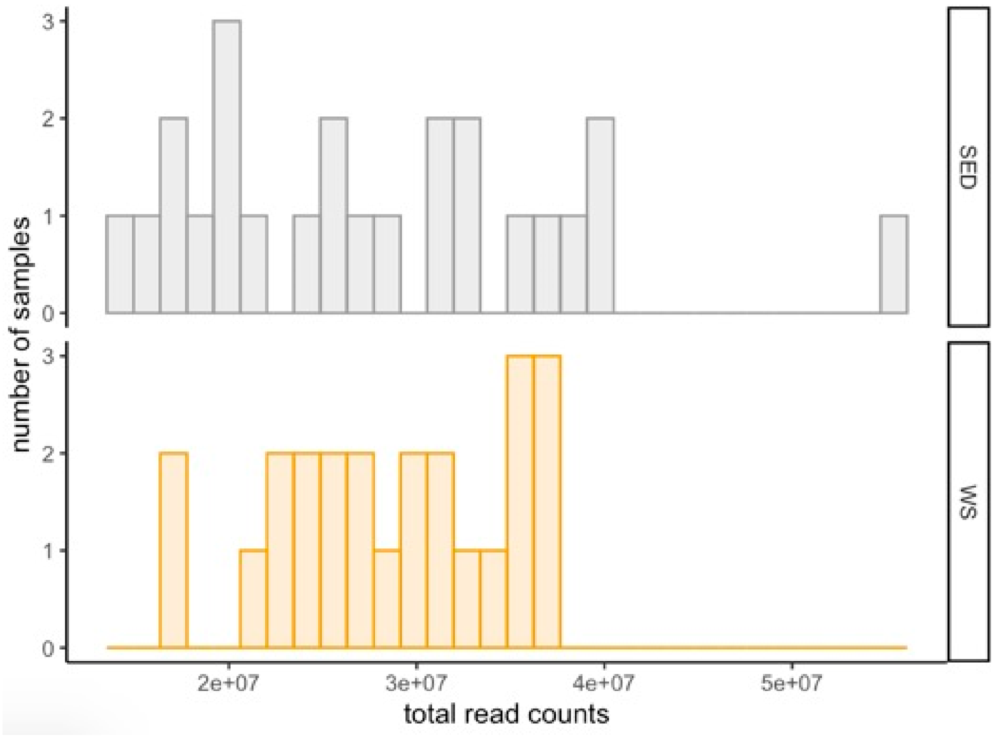

Figure S1. Total read counts for sediment and water samples across the eight collection sites. SED – sediment; WS – water.
